# Effect of thermal treatment of illite on the bioavailability of copper and zinc in the aerobic composting of pig manure with corn straw

**DOI:** 10.3389/fmicb.2024.1411251

**Published:** 2024-06-06

**Authors:** Maia Escobar, Jiaoyang Ji, Yueru Wang, Meiqin Feng, Changjie Bao, Jianxun Ma, Shijia Cui, Sihan Zang, Jinpeng Zhang, Wei Zhang, Guang Chen, Huan Chen

**Affiliations:** ^1^College of Life Science, Jilin Agricultural University, Changchun, China; ^2^Key Laboratory of Straw Comprehensive Utilization and Black Soil Conservation, Ministry of Education, Jilin Agricultural University, Changchun, China; ^3^Key Laboratory of Wetland Ecology and Environment, State Key Laboratory of Black Soils Conservation and Utilization, Northeast Institute of Geography and Agroecology, Chinese Academy of Sciences, Changchun, China

**Keywords:** aerobic compost, illite, bacterial community, pig manure, heavy metal

## Abstract

The large amount of various types of heavy metals in animal manure applied to agricultural field has caused severe threat to the ecosystems of soil environments. In this study, the effect of thermal treatment of illite on the bioavailability of copper (Cu) and zinc (Zn) in the aerobic composting of pig manure with corn straw biochar was investigated. The objectives of this study were to characterize the variations in the bioavailability of Cu and Zn in the aerobic composting of pig manure added with illite treated with high temperatures and to identify the relatively dominant microbes involved in the formation of humus and passivation of heavy metals in pig manure composting based on 16S rRNA high-throughput sequencing analysis. The results showed that in comparison with the raw materials of pig manure, the bioavailability of Zn and Cu in the control and three experimental composting groups, i.e., group I (with untreated illite), group I-2 (with illite treated under 200°C), and group I-5 (with illite treated under 500°C), was decreased by 27.66 and 71.54%, 47.05 and 79.80%, 51.56 and 81.93%, and 58.15 and 86.60%, respectively. The results of 16S rRNA sequencing analysis revealed that in the I-5 group, the highest relative abundance was detected in *Fermentimonas*, which was associated with the degradation of glucose and fructose, and the increased relative abundances were revealed in the microbes associated with the formation of humus, which chelated with Zn and Cu to ultimately reduce the bioavailability of heavy metals and their biotoxicity in the compost. This study provided strong experimental evidence to support the application of illite in pig manure composting and novel insights into the selection of appropriate additives (i.e., illite) to promote humification and passivation of different heavy metals in pig manure composting.

## 1 Introduction

With the rapid development of livestock and poultry industry worldwide, the long-term use of untreated livestock and poultry manure has significantly increased the heavy metal content of farmland soil, causing severe environmental threats to the agricultural ecosystems (Sorathiya et al., 2014; Ravindran et al., 2019). Previous studies showed that about 72–80% of copper (Cu) in pig feed was ultimately excreted with feces, and the amount of zinc (Zn) excreted could be as high as 92–96%, whereas the direct application of untreated pig manure to farmland soil caused increased Cu and Zn concentrations about 10–40 times and 10–25 times higher than those in normal soil, respectively (Bonizzi et al., 1994; Mantovi et al., 2003). Furthermore, plant growth could be inhibited by absorption of heavy metals, subsequently causing the increase in the content of heavy metals in crop seeds and eventually following the food chain to damage the health of human and other animals (Nahm, 2002). The deposition of toxic heavy metals in terrestrial soils may be translocated to plants to cause phytotoxicity, resulting in major concerns to human health via food chain/web (Pathak et al., 2024). Studies have shown that a variety of heavy metals in the soil could migrate to human body through the consumption of vegetables, causing serious risks to human health (Chen et al., 2017; Meng M. et al., 2021; Meng Q. et al., 2021). Therefore, in order to improve the resource utilization of livestock and poultry manure, it is necessary to identify efficient strategies to reduce the bioavailability of heavy metals in the manure. These strategies could facilitate the reduction of the environmental risk caused by the application of organic fertilizer in the agricultural soil, preventing the potential threat of heavy metals in livestock and poultry manure to human health (Kopittke et al., 2008; Hao et al., 2019).

At present, the main strategies to control and prevent the heavy metal pollutions in livestock and poultry manure are to produce the livestock and poultry manure compost (Jeong et al., 2017). In particular, heavy metal passivation agents are added in the process of composting to effectively transform the heavy metals from a high-activity form to a low-activity form, reducing their biological effectiveness (Singh and Kalamdhad, 2013). The advantageous outcomes of this strategy include increased nutrient content, enhanced decay process of composting, and strong passivation of heavy metals (Duan et al., 2019; Wu et al., 2022). For example, the phosphate ore powder is added in the composting process to promote the formation of silicate, carbonate, and hydroxide precipitation of heavy metals, and reduce the biological effectiveness of heavy metals (Cui et al., 2021), while the alkaline additives such as lime could increase the pH level of composting materials, leading to precipitation of carbonate and hydroxide ions generated with heavy metals in the composting materials, ultimately decreasing the biological activity of heavy metals (Wang et al., 2013). In China, fertilizer samples are frequently detected with heavy metals exceeding the limits based on Chinese organic fertilizer standards, e.g., Cr, Cd, and Pb by 13.7, 4.2, 2.4, and 1.4%, respectively (Yang et al., 2017). Furthermore, the application of organic fertilizers containing high levels of heavy metals can lead to excessive accumulation of heavy metals in the soil, resulting in adverse effects on soil quality. For example, studies showed that after 17 years of applying pig manure, the soil concentration of Cd was increased by 18 times (Wu et al., 2012). Moreover, the addition of 4% spent mushroom substrate resulted in a 52.77% decrease in the exchangeable Cd content of the compost and a 65.28% increase in the residual Cd content (Wei et al., 2020). Additionally, bone meal was used as a heavy metal passivator to add to aerobic composting of sludge, causing the proportion of residual Zn, Cr, and Pb increased from 4.71 to 13.65%, 57.53 to 68.77%, and 45.49 to 88.91%, respectively (Li G. et al., 2022; Li S. et al., 2022).

Studies have shown that the use of activated carbon, zeolite, bentonite, and other mineral materials could increase the physical adsorption of heavy metals in composting, due to the large electrostatic force and ion exchange capability as well as the large surface of cavities provided by composting additives (Zhu et al., 2016; Hao et al., 2019). For example, illite is commonly used as composting additive due to its convenient availability, low cost, strong adsorption capacity, and high cation exchange capability (Meng Q. et al., 2021). Studies have shown that illite is rich in potassium, which could be processed to produce potassium nitrogen fertilizer and K_2_O solution for easy absorption by plants, providing enhanced functions of potassium fertilizer (Szczerba et al., 2020; Meng M. et al., 2021; Meng Q. et al., 2021). In general, the thermal treatment (i.e., burning) is used to remove the water and surface impurities, activate the adsorption center, and improve the adsorption capacity of illite (Zhu et al., 2016; Fox et al., 2019; Pan et al., 2021; Mills et al., 2023).

Due to the non-biodegradability of heavy metals, pollution from heavy metals has become an environmental issue that needs to be urgently solved worldwide (Chen et al., 2015). Previous study showed that inoculation of white rot fungi during sludge composting reduced the bioavailability of several heavy metals (Zhang et al., 2018). Both montmorillonite and illite are layered clay minerals in structure. Montmorillonite is commonly used as a passivator for heavy metals in composting. For example, montmorillonite is added as a passivator in aerobic composting of chicken manure to significantly reduce the bioavailability of Cu and Zn, decreased by 81.2 and 15.6%, respectively (Hao et al., 2019), while both illite and montmorillonite were introduced into cattle manure compost, showing significant influence on the degradation of lignocellulose during the composting process (Meng M. et al., 2021; Meng Q. et al., 2021).

In this study, the effects of thermal treatment of illite on the bioavailability of two heavy metals (i.e., Cu and Zn) and the composition of bacterial communities were investigated to explore the biotic and abiotic mechanisms underlying the passivation of heavy metals. This was the first investigation using illite in the compositing process of pig manure with corn straw biochar to improve the passivation of heavy metals. The advantages of using illite in the composting process of pig manure included effective passivation of heavy metals and enhanced composting process of pig manure, as observed in the physiochemical and 16S rRNA sequencing analyses. The selection of these two heavy metals was due to the presence of toxic metals in pig manure, especially Cu and Zn, and the issue was more severe compared to other livestock manures. Previous studies showed that Cu exceeding the standard primarily occurs in the feed of pigs, while Zn exceeding the standard is present in nearly all feed samples (Wang et al., 2013). The goals of this study were: (1) to characterize the variations in the bioavailability, i.e., passivation of Cu and Zn in the aerobic composting of pig manure added with illite treated with two high temperatures (200 and 500°C), and (2) to identify the relatively dominant microbes involved in the formation of humus (i.e., metabolism of humic acid) and passivation of heavy metals in pig manure composting based on 16S rRNA high-throughput sequencing analysis. The results provided novel insights into the selection of appropriate additives to promote humification and passivation of different heavy metals in pig manure composting.

## 2 Materials and methods

### 2.1 Design of composting system

The pig manure composting was performed in foam boxes each of 354 mm (length) × 254 mm (width) × 271 mm (height) with wall thickness of 27 mm. The pig manure materials were collected from the commercial farm near Changchun, Jilin Province, China. Corn straw was provided by the Key Laboratory of Straw Comprehensive Utilization and Black Land Protection, Jilin Agricultural University (Changchun, China), dried, and ground into particles of about 1.5 cm^3^ in size. The pig manure and corn straw were mixed for composting based on carbon/nitrogen (C/N) ratio of 25–30, with the initial moisture content adjusted to about 60%. The corn straw was added based on the carbon/nitrogen ratio (in the range of 25–30) of pig manure. This ratio fell into the ranges commonly used in the previous studies (Yu et al., 2020). This information has now been added in the Section 2.1 of Materials and Methods of our revised manuscript. A total of four groups of experiments were set up as follows: the control group (CK) contained the pig manure and corn straw and three treatment groups, i.e., group I contained the pig manure and corn straw added with untreated illite, and groups I-2 and I-5 contained the pig manure and corn straw added with illite thermally treated under 200 and 500°C, respectively. The selection of these two temperatures is based on the previous studies (Konan et al., 2012). Previous studies showed that although heating above 450°C cannot affect the structure of illite, the number of edge-accessible Lewis acid sites on illite is significantly reduced, which would impact its ability to bind with heavy metal ions in composting, ultimately affecting the passivation effectiveness of illite. Furthermore, considering economic and energy-related factors, the thermal treatment of illite was performed using 200 and 500°C, respectively (Konan et al., 2012). The addition of corn straw was used to adjust the carbon/nitrogen ratio during the composting process of pig manure. The high carbon content in corn straw effectively increased the carbon/nitrogen ratio of the entire composting material. Therefore, corn straw was added for co-composting with pig manure, and the initial carbon/nitrogen ratio was adjusted to the range of 25–30 in order to accelerate composting process. Samples of composting materials were collected from the top, middle, and bottom layers of the compost heap on days 0, 1, 3, 7, 14, 21, 28, and 35, respectively. Each sample was divided into two parts, with one part air-dried in the shade and then filtered through 80-mesh filters for subsequent analysis of heavy metals and physicochemical properties, and the other part stored in a refrigerator at −80°C for 16S rRNA high-throughput sequencing analysis to investigate the composition of microbial communities.

### 2.2 Analysis of physicochemical properties and heavy metals

The temperature of the compost was measured daily at the center of each compost pile using digital thermometers, with the pH and electrical conductivity (EC) measured using pH meter (PHS-25, INESA Scientific Instrument Co., Ltd., Shanghai, China) and the EC meter (DDS-307A, INESA Scientific Instrument Co., Ltd., Shanghai, China), respectively, based on the water: sample ratio of 10:1 (v/w), shaken for 2 h prior to measuring (Cui et al., 2021; Zhan et al., 2022). The content of total organic carbon (TOC) was measured using the Potassium dichromate oxidation method and the total nitrogen content was determined by Kjeldahl method, respectively (Bremner, 1965).

The extractions of humus, fulvic acid (FA), and humic acid (HA) were performed as previously described (Shi et al., 2020). The level of decay during composting was evaluated by two decay indices, i.e., the percentage of humic acid in the total humus (Pha) and the ratio of HA and FA contents (HA/FA), indicating the polymerization rate of HA (Kumada et al., 1967; Roletto et al., 1985).

The total contents of Cu and Zn were determined by inductively coupled plasma optical emission spectroscopy (ICP-OES) (Agilent 7,900, USA) after aqua regia digestion. The extraction of heavy metals was performed following the modified BCR method, generating four different forms based on the level of exchangeability (Exc), reduction (Red), oxidability (Oxi), and residues (Res) (Wu et al., 2021), i.e., Cu and Zn detected in these four forms were abbreviated as ExcCu, RedCu, OxiCu, and ResCu, and ExcZn, RedZn, OxiZn, and ResZn, respectively (Rauret et al., 1999). The potential ecological risk index (RI), contamination factor (*C_f_*), and ecological risk factor (Er) of both Cu and Zn were further evaluated as previously reported (Hakanson, 1980; Ngole-Jeme et al., 2017) (Eqs. 1–3).


(1) $$C_{f}=\frac{C_{i}}{C_{n}}\text{,}$$

(2) $$E_{r}={TrC}_{f}\text{,}$$

(3) $$RI=\overset{n}{\underset{i}{\sum }}Er\text{,}$$

where Ci was the concentration of non-residual component of the heavy metal (i.e., exchangeable + reduction + oxidability) in composting, Cn represented the concentration of the stable component of the heavy metal (residues), and Tr indicated the toxicity coefficient of the heavy metal.

### 2.3 Microbiological analysis

The bacterial community structure was analyzed by high-throughput sequencing of the bacterial 16S rRNA V4 region based on compost samples collected on 0, 3, and 35 days of both control and treatment groups. Genomic DNA was extracted using soil and fecal genomic DNA extraction kit (TianGen, Beijing, China) and PCR-amplified using primers 515F (5’-GTGCCAGCMGCCGCGGTAA-3′) and 806R (5’-GGACTACHVGGGTWTCTAAT-3′) to obtain the 16S rRNA fragments in the samples. The sequencing library construction was performed using the NEB Next➅ Ultra™ II FS DNA PCR-free Library Prep Kit (New England Biolabs, Beijing, China). After the library quality control, PE 250 sequencing was performed by NovaSeq 6,000 and quality control analysis was performed using the standard method of the NovaSeq sequencing platform (Novogene, Beijing, China). Amplicon Sequence Variants (ASVs) were obtained using DADA2 of QIIME2 (Version QIIME2-202202) to filter the effective tags. Taxonomic annotation was performed by QIIME2 based on Silva 138.1, with multiple sequences aligned, phylogenetic trees constructed, and microbial relative abundance calculated using the SVG function.

### 2.4 Statistical analysis

Statistical analyses were performed using Office 2021 and SPSS 26.0 was used to evaluate the significant differences among groups based on *p* < 0.05. Graphs were generated using Origin 2021. The R package (version 4.0.2) was used to evaluate the relationships among microbial taxa, perform the non-metric multidimensional scaling analysis (NMDS) and redundancy analysis (RDA), generate heatmaps of bacterial communities, and both alpha diversity indices and principal coordinate analysis (PCoA) based on the Bray-Curtis distance.

## 3 Results and discussion

### 3.1 Effect of illite on physiochemical properties of the compost heap

**Figure 1:**
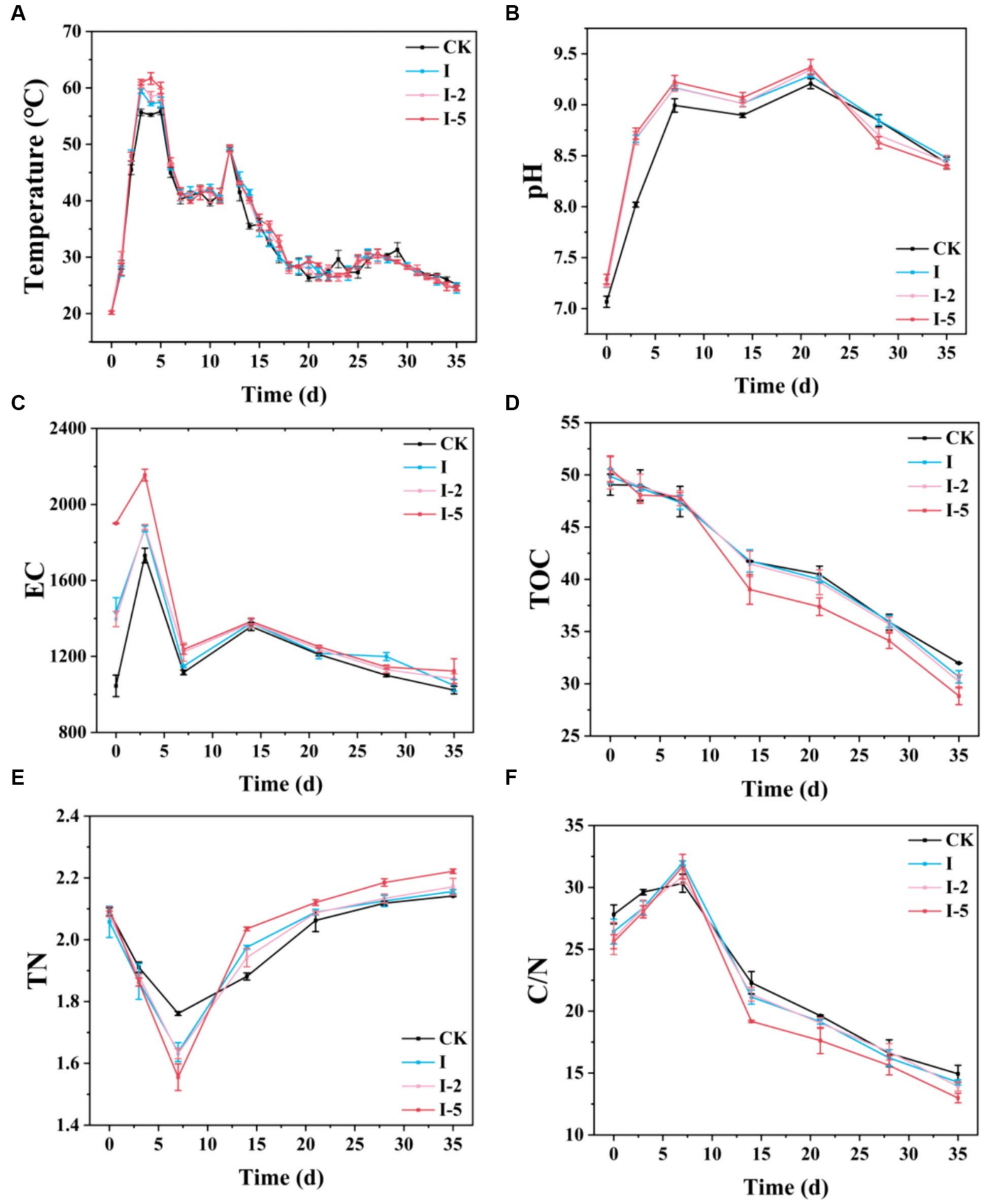
Temporal variations in **(A)** temperature, **(B)** pH level, **(C)** electrical conductivity (EC), **(D)** total organic carbon (TOC) content, **(E)** total nitrogen (TN)content, and **(F)** carbon/nitrogen (C/N) ratio in four groups of composting samples, i.e., control (CK) group and treatment groups I, I-2, and I-5, respectively.

Temperature in the compost is an important indicator of the microbial activity and degradation of organic matters in the composting process. Our results revealed the variations in the temperatures of compost of different groups (Figure 1A). The process of composting was generally divided into three stages, i.e., heating, high temperature, and decay periods, showing largely the congruent variation patterns among the different groups of compost. In particular, the temperature of each group was initially increased rapidly during the heating period (day 1) and reached the high temperature period (> 45°C) on day 2, which lasted until day 6. The highest temperatures of the treatment groups were all significantly higher than that of the control group, with the highest temperature (61.66°C) observed in the I-5 group, followed by the groups of I-2 (60.5°C), I (59.43°C), and CK (55.83°C), respectively. These results suggested that compared with the CK group, thermal treatment resulted in high porosity of illite, maintaining more oxygen in the compact to effectively improve the activity of aerobic microorganisms, accelerate the degradation of organic matters, and then increase the temperature of the compact. These results were consistent with those previously reported (Wang et al., 2016). In 7–10 days, the temperature in the compost pile was rapidly decreased to 39.66–42.33°C, mainly due to the gradual decrease of the contents of degradable organic matters. From 11 to 13 days, the compost turning was performed to increase the aeration of compost, and secondary heating phenomenon was observed in all groups of compost, as previously reported (López-González et al., 2015). The temporary temperature increase to 49.16–49.33°C was probably due to the enhanced contact of the refractory organic matters in the compost heap with oxygen, accelerating the reduced degradation caused by organic consumption and the recovery of thermophilic microbial activity (Petric et al., 2009; Zhao et al., 2010).

The similar patterns were observed in the variations of pH levels of the compost under different treatments (Figure 1B). In the first 7 days of composting, as the temperature was increased, the pH levels were rapidly increased in each treatment, mainly due to the degradation of the nitrogen-containing organic matters in the compost, producing a large amount of NH_3_, which was dissolved in water to increase the pH levels to 8.99–9.22 in the compost heap. In 14 days, the pH levels were slightly decreased to the range of 8.89–9.07, then increased to the highest levels of 9.20–9.37 in 21 days, and finally gradually decreased to the range of 8.39–8.44 in 35 days, with the maximum pH reduction observed in the I-5 group (pH = 8.39). These results showed that the pH level reduction in the illite-treated groups was significantly faster than that of the CK group, with each compost heap meeting the weak alkaline requirement of mature composting (Tiquia and Tam, 2000). In the later stage of composting, the fast decline of pH levels in the illite-treated groups was probably due to the high porosity of illite. These findings were consistent with the results previously reported, showing that additional minerals such as illite mixed in the compost elevated the pH level, improved the microbial activity to further promote the degradation of lignocellulose, and accelerated the production of organic acids and nitrification to increase the generation and accumulation of nitrate nitrogen (Kopeć et al., 2013, 2014; Meng M. et al., 2021).

Due to its close association with the concentration of soluble salt in organic fertilizer, EC is used as an important indicator to measure the content of soluble salt in organic fertilizer. Our results showed that at the beginning of the composting process, the EC in the treatment groups was increased as the composting time was increased compared with the control group. In 3 days, the EC in each treatment group reached their highest levels, with the highest EC value revealed in the I-5 group among the three treatment groups (Figure 1C). At day 3, the highest levels of EC reached 1357.33 ± 21.36 μS/cm, 1,375 ± 21.93 μS/cm, 1375.33 ± 24.11 μS/cm, and 1384.33 ± 17.47 μS/cm in the groups of control, I, I-2, and I-5, respectively. The rapid increase in EC in the early stage of composting could be attributed to the production of a large number of soluble ions caused by the active biological activities of microbes (Tang et al., 2020; Abdi et al., 2023). After 3 days composting, the EC was rapidly decreased, probably because of the reduced microbial activity caused by water loss at high temperature. In 14 days, after the compost turning was performed, the accelerated metabolic activity caused a transient increase in the EC. Then, the EC continued to decrease to the end of the experiments in 35 days, which was mainly related to the slow metabolic activity, ammonia volatilization, and precipitation of mineral ions, as previously reported (Ge et al., 2020; Zhang et al., 2023).

The contents of TOC in different treatments were significantly decreased as the time of composting was increased (Figure 1D). In the early stage of composting, the reduction of TOC content was slow, and the reduction rate reached the highest levels in 7–14 days, which was probably caused by the rapid degradation of substances with low molecular weights, such as lipids, proteins, and sugars, as previously reported (Chen et al., 2021; Aydın, 2023). The TOC reached the lowest levels toward the end of experiment, i.e., the TOC was decreased to 31.98% ± 0.49, 30.66% ± 0.59, 30.21% ± 0.64, and 28.83% ± 0.84 in the groups of control, I, I-2, and I-5, respectively, at day 35. During the decay period, the concentrations of organic matters were not significantly changed and the materials that were easy to degrade became exhausted, while the materials that were relatively difficult to degrade, i.e., cellulose, hemicellulose, and lignin, became the main components of organic composition, and the organic matters in the compost pile were stabilized and difficult to be degraded and utilized by microbes (Liu et al., 2008; Devi and Khwairakpam, 2023). Therefore, the TOC degradation in each group became slow in 14 days. Furthermore, the different variation patterns from those of TOC content were revealed in TN content over time (Figure 1E). In particular, the TN content was rapidly decreased to the lowest level in 7 days and then gradually increased to about 2.1–2.2 g/kg of each group. Compared with the control group, less nitrogen loss was observed in the illite treatment groups, probably due to the reduction of organic matter and release of CO_2_ during the composting process. At the beginning of composting, nitrogen in untreated manure was mainly composed of organic nitrogen. During the composting process, organic nitrogen was decomposed into ammonium nitrogen by microbial action, resulting in the generation and release of ammonia. In the early stages, the fastest rate of decrease was detected in group I-5. From the 7th day until the end of composting, due to the substantial decomposition of organic matter, the level of TN was gradually increased in all groups. At the end of composting, the TN content in each group was around 2.1 to 2.2. Compared to other groups, the I-5 treatment group showed the highest TN content at the end of composting, indicating less nitrogen loss in its composting pile. The final C/N ratios of different treatments were detected in the range of 12.9–14.99, which met the national standards (GB 7959–1987) for returning organic fertilizer to the field in China (Zhou et al., 2015). From 1 to 7 days of the composting process, the C/N ratio of each group was increased (Figure 1F). The contents of carbon and nitrogen of illite treatment groups were higher than those of the control group in the high temperature period, probably due to the active microbial growth and high microbial diversity, as previously reported (Qiu et al., 2019). Then, the C/N ratio was rapidly decreased from 7 to 14 days and slowly decreased to the end of the experiments, which was controlled by the degradation rate of carbon and nitrogen. The similar variation patterns were detected in the previous studies (Yang et al., 2013).

### 3.2 Effect of illite on humus content of the composting products

**Figure 2:**
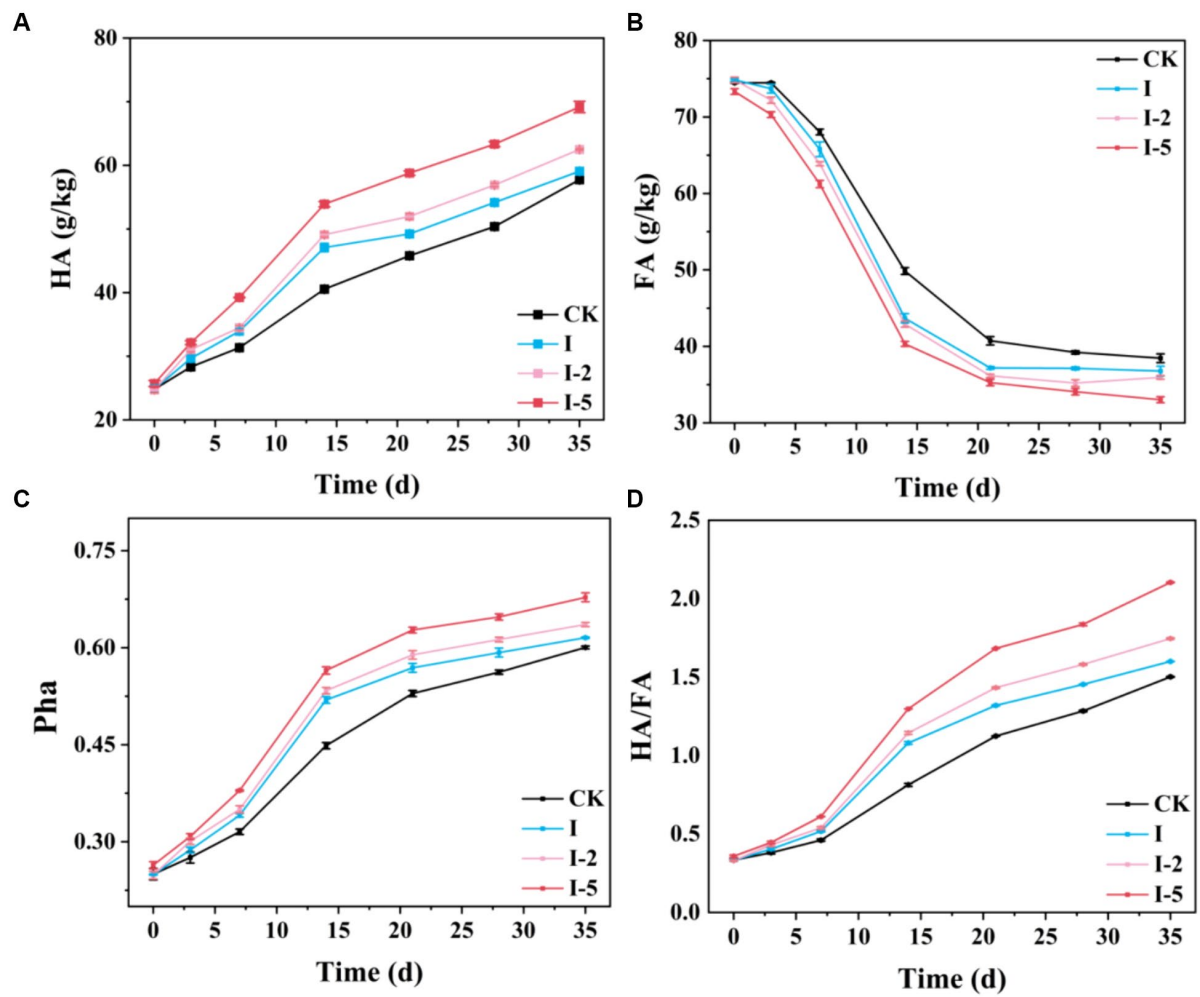
Temporal changes in **(A)** humic acid (HA) content, **(B)** fulvic acid (FA) content, **(C)** percentage of HA (Pha), and **(D)** HA/FA ratio during composting in four groups of samples, i.e., control (CK) group and treatment groups I, I-2, and I-5, respectively.

In the process of composting, i.e., humification of organic matters, the changes in humus content indicate the progress and degree of composting (Rawoteea et al., 2017). The humus matters, as the important components of organic matters, with the ability of adsorption and chelation with heavy metals, are mainly composed of HA and FA (Li et al., 2023; Liu et al., 2023). Our results showed that in 14 days after the composting, the HA content was rapidly increased in each treatment group, showing the highest rates of organic matter degradation and humification, and then slowly increased, reaching the maximum level at the end of the composting process (Figures 2A,B). The HA content was constantly the highest in the I-5 group during the composting process, reaching the highest level of 69.16 g/kg in 35 days. The HA contents of CK, I, I-2, and I-5 groups were increased by 32.92, 34.23, 37.67, and 44.34 g/kg, respectively, compared with the raw material. These results suggested that addition of illite could promote the generation of HA, which was mainly converted from FA by microorganisms in the compost heap, while FA could be easily degraded in the process of composting. Therefore, the content of FA was rapidly decreased in the early composting stage and eventually tended to be stabilized, as observed in the variations in the FA contents, showing the highest reduction rate of FA in the I-5 group. In 35 days, the FA contents in CK, I, I-2, and I-5 groups were decreased to 38.45, 36.77, 35.94, and 33.03 g/kg, respectively, probably caused by the addition of illite promoting the degradation of organic matters in the early stage of composting. These findings were consistent with those previously reported (Awasthi et al., 2017).

Due to the degradation of organic matters in the composting process, the raw materials of composting were concentrated, making it difficult to accurately reflect the humification process based on only the humus concentration changes. Therefore, both the Pha and the HA/FA ratio were investigated to further assess the decay degree of the compost heap (Figures 2C,D), i.e., the higher HA/FA ratio indicated the higher humification degree. The results showed that both the ratio of HA/FA and the Pha were increased throughout the entire composting process, with the rapid increases observed from 7 to 14 days, indicating that the maximum rate of organic matter degradation and humification were obtained in the high temperature period of the composting process (Fourti et al., 2010). In 35 days, the HA/FA ratios reached 1.50, 1.59, 1.74, and 2.1 in the CK, I, I-2, and I-5 groups, respectively, indicating that all compost heaps were fully decayed (HA/FA ratio > 1) (Roletto et al., 1985). In 35 days, the Pha in the CK, I, I-2, and I-5 groups reached 0.60, 0.61, 0.63, and 0.67, respectively. Both the Pha and HA/FA ratio in I-5 group were constantly the highest among all groups, indicating the highest stability and humification levels of the organic matters in I-5 group.

### 3.3 Variations in the contents and forms of Cu and Zn

The variations in the total amount of Cu and Zn in different composting groups were investigated (Table 1). The results showed that the total amount of Cu and Zn was successively increased as the composting time was increased in the temporal order of 0 d > 3 d > 35 d. In 35 days, the concentrations of Zn and Cu were significantly higher than those of the control group, which was attributed to the reduction of organic matters and the release of CO_2_ in the composting process, resulting in relatively more concentrated heavy metals, as previously reported (Wang et al., 2019). It was noteworthy that an increase in the total amount of heavy metals in the compost heap could not necessarily suggest an enhanced detrimental effect of the heavy metals, as the heavy metal mobility and bioavailability are more important than the total amount of heavy metals in evaluating the compost products.

**Table 1:** Variations in the contents of Cu and Zn in 0, 3, and 35 days of four composting groups, i.e., the control (CK) group and three treatment groups I, I-2, and I-5, respectively (*n* = 3).

Sample	Time (d)	Zn (g/kg)	Cu (g/kg)
CK	0	695.55^a^ ± 8.83	202.21^b^ ± 4.12
	3	929.79^a^ ± 9.89	235.72^c^ ± 0.98
	35	1024.47^a^ ± 48.43	276.35^c^ ± 4.02
I	0	695.56^a^ ± 5.01	211.59^ab^ ± 2.97
	3	936.58^a^ ± 28.38	246.20^b^ ± 3.53
	35	1044.33^a^ ± 50.39	290.72^b^ ± 4.11
I-2	0	621.43^b^ ± 24.52	219.54^a^ ± 8.57
	3	888.45^b^ ± 6.84	260.05^a^ ± 7.38
	35	1063.08^a^ ± 24.50	219.52^ab^ ± 1.50
I-5	0	610.75^b^ ± 30.63	206.11^a^ ± 10.13
	3	879.90^b^ ± 7.99	262.30^a^ ± 2.12
	35	1055.18^a^ ± 46.27	306.76^a^ ± 9.43

Data are presented as mean ± standard deviation (SD). Different superscript letters in each column indicate the significant difference among the contents of different groups at the same composting time (*p* < 0.05).

**Figure 3:**
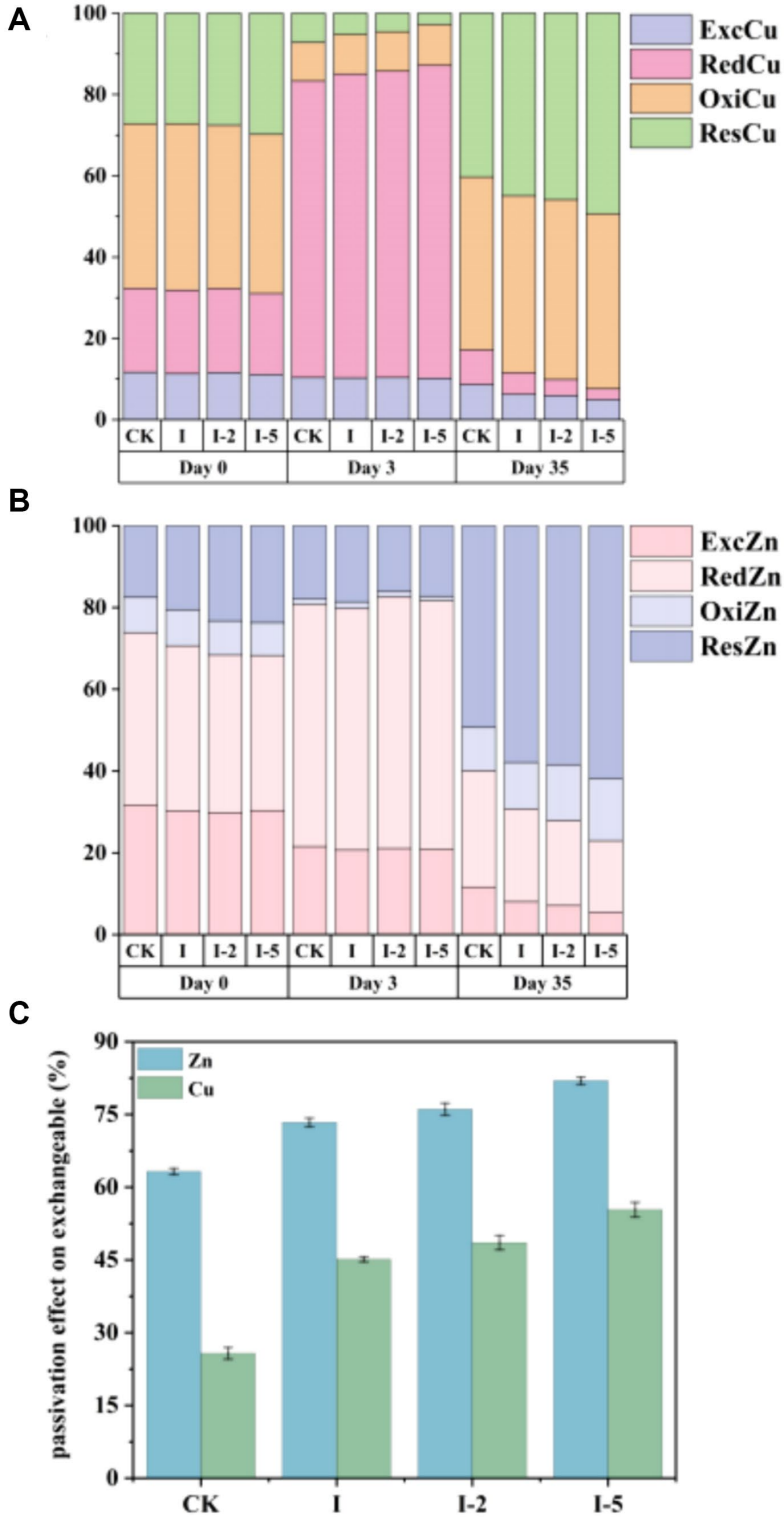
Relative distributions of four different forms of **(A)** Cu and **(B)** Zn in the control (CK) and three treatment groups (i.e., I, I-2, and I-5) on days 0, 3, and 35 of the composting process, and **(C)** the passivation rates of exchangeable Zn and Cu in CK, I, I-2, and I-5 groups of composting on day 35.

The changes of various forms of heavy metals play a crucial role in the quality and toxicity of compost. The relative distributions of different forms of Cu and Zn were investigated (Figure 3). The results showed that in the initial stage of composting, the biologically effective components (i.e., the exchangeable and the reducible components) accounted for a high proportion in the total amount of Zn (over 60%), whereas Cu mostly existed in the form of non-biological effective components (i.e., oxidable and residual components) (over 60%). As the time of composting was increased, the biologically effective components in Zn and Cu were decreased. In 35 days, the relative proportion of biologically effective components (i.e., bioavailability) in Cu and Zn was significantly lower in the treatment groups than that of the control group. Due to the abundant functional groups on the surface of illite, illite readily forms complexes with heavy metals through interaction, thereby promoting the reduction of bioavailable fractions of heavy metals (Meng M. et al., 2021; Meng Q. et al., 2021).

In the high temperature period of composting, the main component of Cu existed in RedCu. In 35 days, the contents of RedCu were decreased by 64.61, 74.23, 80.92, and 86.81%, and the contents of ExcCu were decreased by 36.08, 44.31, 48.45, and 55.95%, in the CK, I, I-2, and I-5 groups, respectively. In groups I, I-2, and I-5, the contents of OxiCu were increased by 5.71, 5.12, and 7.6%, respectively, compared with the CK group. In the early stage of compositing, Zn mainly existed in ExcZn, which was decreased by 62.08, 73.02, 77.11, and 81.67% in the CK, I, I-2, and I-5 groups, respectively, during the decay period of compositing. The content of reduced form of Zn was mostly decreased as the composting time was increased. Compared with the CK group, the contents of RedZn in groups I, I-2, and I-5 were decreased by 12.09, 15.39, and 22.64%, respectively. These results showed that group I-5 was observed with the highest passivation effects for Zn of both forms of ExcZn and RedZn. These results were probably caused by the enhanced formation of the coordination bond between Zn and organic matters, i.e., the increased specific surface area caused by high temperature treatment exposed more cations in the interlayer of illite to interact with the heavy metal ions in the compost. These results were consistent with those previously reported (Beesley et al., 2010). Compared with the CK group with the passivation effect of Cu (36.08%) and Zn (62.08%), the passivation effects of ExcCu and ExcZn were enhanced in each treatment group, showing higher passivation effects of Cu (44.31%) and Zn (73.02%), Cu (48.45%) and Zn (77.11%), and Cu (55.93%) and Zn (81.67%) in I, I-2, and I-5 groups, respectively. These results indicated that the illite treatment groups obtained higher passivation effects of Cu and Zn in the composting process than that of control group, and the contents of ExcZn and ExcCu could be effectively reduced by heat, i.e., increased temperature in the compost pile. Ultimately, the bioactivity of Cu and Zn was generally reduced in the pig manure composting process, thus inhibiting the bioavailability of both Cu and Zn. Similarly, previous studies have shown that the addition of adsorbents, such as biochar, bentonite, and phosphate-solubilizing bacteria, to the aerobic composting, could significantly modulate the bioactivity and toxicity of heavy metals (Li et al., 2020). In summary, Zn and Cu in composting materials have high bioavailability. After composting, pig manure can effectively reduce the mobility, toxicity, and bioavailability of these heavy metals in manure, causing a transformation of Zn and Cu from a biologically available state to a non-biologically available state. These results may be associated with the enhanced formation of coordination bonds between heavy metals and organic matter. It is possible that the high-temperature treatment of illite increased its specific surface area to expose more interlayer cations of illite. As a result, the heavy metal ions in the compost underwent exchange reactions with the interlayer cations.

The results of RI, C_f_, and Er of both Cu and Zn in the composting process showed that at the end of composting, the Er of Cu and Zn was decreased to less than 40%, the *C_f_* of Cu and Zn was decreased to <4 and < 1, respectively, and the RI of Cu and Zn in the heap was decreased to below 50 (Table 2). These results showed that the addition of illite caused a great impact on the ecological risk of Cu and Zn in pig manure composting. Previous studies showed that the addition of minerals reduced the ecological risk of composting by regulating the bacterial structure of microbial community in the compost (Li G. et al., 2022; Li S. et al., 2022).

**Table 2:** Variations in contamination factor (*C_f_*), ecological risk factor (Er), and potential ecological risk index (RI) of Cu and Zn in the control (CK) and three treatment groups (i.e., I, I-2, and I-5) of composting (*n* = 3).

Group	Time (d)	Cu		Zn		RI
		C_f_	Er	C_f_	Er	
CK	0	2.69 ± 0.36	13.34 ± 1.83	4.71 ± 0.18	4.71 ± 0.18	18.05 ± 1.90
	35	1.48 ± 0.10	7.40 ± 0.52	1.02 ± 0.01	1.02 ± 0.01	8.42 ± 0.52
I	0	2.67 ± 0.12	13.34 ± 0.62	3.85 ± 0.03	3.85 ± 0.03	17.19 ± 0.6
	35	1.22 ± 0.02	6.13 ± 0.11	0.72 ± 0.02	0.72 ± 0.02	7.95 ± 0.12
I-2	0	2.63 ± 0.18	13.15 ± 0.93	3.28 ± 0.68	3.28 ± 0.68	16.43 ± 0.76
	35	1.17 ± 0.07	5.89 ± 0.37	0.71 ± 0.02	0.71 ± 0.02	6.59 ± 0.37
I-5	0	2.34 ± 0.26	11.74 ± 1.33	6.20 ± 0.54	6.20 ± 0.54	17.94 ± 1.83
	35	1.02 ± 0.04	5.12 ± 0.24	0.61 ± 0.04	0.61 ± 0.04	5.72 ± 0.20

Data are presented as mean ± standard deviation (SD).

### 3.4 Variations in the taxonomic composition of microbial communities during composting

The changes of alpha diversity index in different periods of composting in each group are shown in Table 3.The Coverage index reached above 0.99, which indicates that the results of this sequencing can truly reflect the changes of bacterial communities in the samples. During the composting process, the Chao1 index of each treatment group showed an overall decreasing trend, reaching the lowest value at the end of composting. At the end of composting, the Chao 1 index of each group of compost reached the lowest value. At the early stage of composting each pile was rich in bacterial species, and due to the death of a large number of pathogenic bacteria in the pile during the high temperature period, some microorganisms went into a dormant or dead state. At the end of composting the Chao1 index was lower in all I-5 treatment groups than in other groups. Both Shannon index and simspon index showed a decreasing and then increasing trend, which indicated that heat-treated illite could enhance the dominant role of the dominant flora in the heap. The addition of illite can change the original microbial community structure in the compost, increase the diversity of compost groups, and strengthen the abundance of dominant bacteria.

**Table 3:** Changes in alpha diversity index across different periods in the control (CK) and three treatment groups (i.e., I, I-2, and I-5) of composting (*n* = 3).

Sample	Time	Chao1	Shannon	Simpson	Coverage
CK	Day 0	1078.484 ± 34	7.402 ± 0.76	0.965 ± 0.006	0.998
I	Day 0	1032.346 ± 28	7.406 ± 0.42	0.972 ± 0.005	1.000
I-2	Day 0	1031.091 ± 74	7.396 ± 0.32	0.976 ± 0.005	1.000
I-5	Day 0	1028.164 ± 51	7.336 ± 0.75	0.982 ± 0.007	0.996
CK	Day 3	764.524 ± 106	6.690 ± 0.61	0.946 ± 0.003	0.998
I	Day 3	779.146 ± 63	6.572 ± 0.51	0.931 ± 0.005	0.996
I-2	Day 3	782.827 ± 71	6.560 ± 0.13	0.936 ± 0.003	0.994
I-5	Day 3	783.124 ± 42	6.313 ± 0.42	0.950 ± 0.007	0.996
CK	Day 35	754.611 ± 38	7.404 ± 0.67	0.972 ± 0.004	1.000
I	Day 35	741.760 ± 39	7.443 ± 0.23	0.965 ± 0.005	1.000
I-2	Day 35	703.291 ± 57	7.452 ± 0.70	0.981 ± 0.004	1.000
I-5	Day 35	693.209 ± 63	7.483 ± 0.31	0.983 ± 0.006	1.000

Data are presented as mean ± standard deviation (SD).

**Figure 4:**
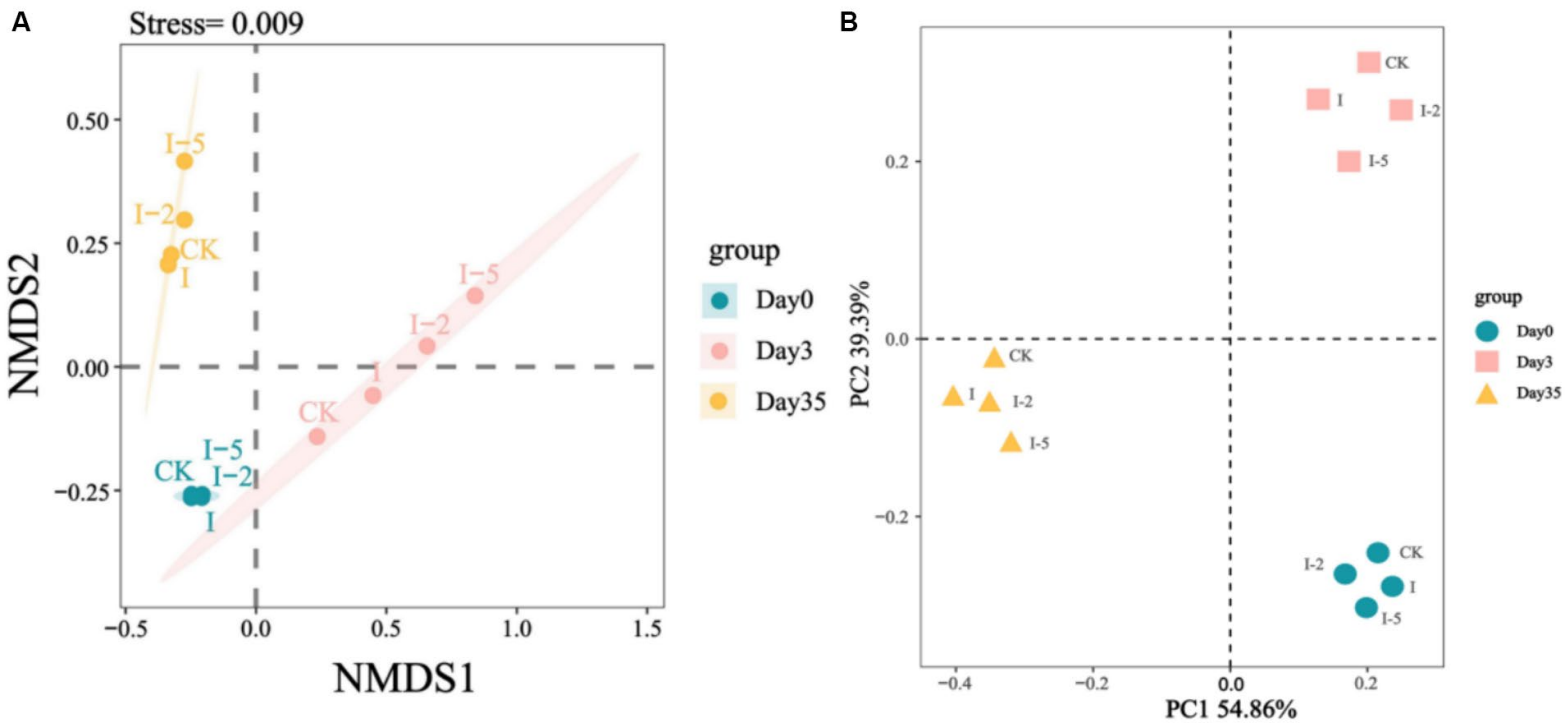
Variations in the diversity of microbial communities in the control (CK) and three treatment groups (i.e., I, I-2, and I-5) during composting based on NMDS analysis **(A)** and PCoA analysis **(B)**.

With the changes in the composting microenvironment and the degradation of organic matters, significant alterations were revealed on the succession of the microbial communities. Figure 4 showed the NMDS analysis and PCoA analysis of species for each treatment in each period of composting, respectively. According to Figure 4A, it can be seen that the community structure of bacteria in each period was obviously separated, in which the Stress value was 0.009, which well proved the reliability of the data, and the experimental samples could truly reflect the bacterial community composition of different compost treatments. With the fermentation of compost, the samples collected in different periods were roughly distributed in three different quadrants, which were far away from each other, especially the high-temperature period was far away from the warming period and the putrefaction period, respectively, and there were obvious differences in the structure of the bacterial communities in various stages of composting. The PCoA plot also showed that the microbial communities were mainly clustered according to the changes in composting time. The horizontal and vertical coordinates were PC1 (54.86%) and PC2 (39.39%), especially the community structure of the high-temperature period differed from that of other periods; the samples of the treatment groups in the high-temperature period were far apart, which indicated that the bacterial community of the samples in the high-temperature period was more different.

**Figure 5:**
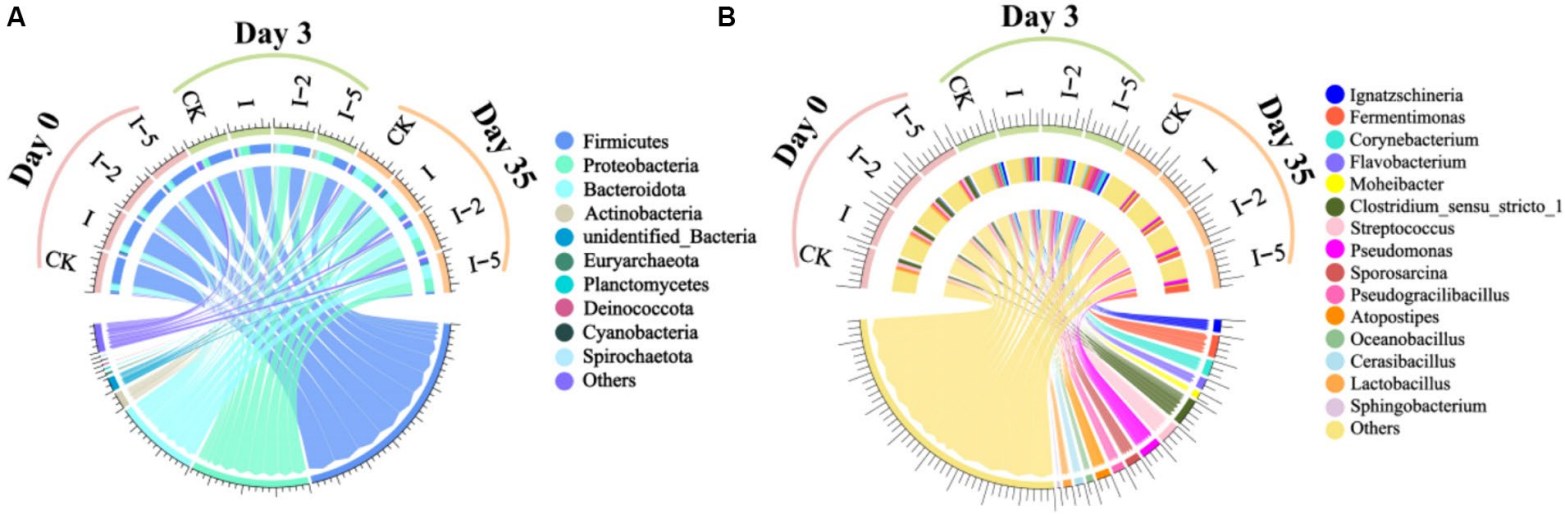
Taxonomic compositions at the phylum **(A)** and genus **(B)** levels on days 0, 3, and 35 in the control (CK) and three treatment groups (i.e., I I-2, and I-5), respectively.

The microbial compositions were characterized at both phylum and genus levels to investigate the relative abundance of bacteria in different composting groups on days 0, 3, and 35, respectively (Figures 5A,B). The results showed that at the phylum level, the relatively dominant phyla included Firmicutes, Actinobacteria, Proteobacteria, and Bacteroidetes, accounting for more than 90% of the total bacterial taxa. In 3 days, the relative abundance of Firmicutes was significantly reduced in each treatment group, and the relative proportions of Proteobacteria and Actinobacteria, which were revealed to play crucial roles in organic matter decomposition and carbon cycle (Zhu et al., 2021), were gradually increased. It is noteworthy that although many taxa of Firmicutes are pathogens causing various human diseases, the pathogenic microorganisms of Firmicutes that are not resistant to high temperature would die during the high temperature period of composting (Subirats et al., 2022). As the bacterial phylum actively involved in nitrogen conversion, *Proteobacteria* participated in nitrogen conversion during the high temperature period of all composting groups, and the relative abundance of *Proteobacteria* was significantly increased during the high temperature period, with the highest relative abundance identified in the I-5 group. These results were consistent with those previously reported (Lu et al., 2023). At the end of composting process, the relatively dominant phyla included both Proteobacteria and Bacteroidota, both playing an important role in carbon cycling and nitrogen mineralization and showing significantly higher relative abundances in the treatment groups than that in the control group. At the genus level, the pathogenic genera *Ignatzschineria* and *Streptococcus* showed higher relative abundances during the early composting period in the treatment groups. Due to their intolerance to heat, the relative abundances were greatly reduced during the high temperature period and decreased to the lowest relative abundance in the decay period. These results indicated that the addition of illite in the composting process effectively inhibited the growth and survival of *Streptococcus* (with low relative abundance <0.01%) in the mature compost. These results were consistent with those previously reported (Coelho et al., 2022; Maniam and Argentine, 2022). *Fermentimonas* was revealed with the highest relative abundance at the end of the composting process, which was probably associated with the degradation of glucose and fructose and involved in heavy metal passivation, showing the highest relative abundance at the end of composting of group I-5. These results were consistent with those previously reported (Duan et al., 2022). Both genera *Corynebacterium* and *Cerasibacillus* showed the highest relative abundances in each treatment group during the high temperature period of composting. These results were consistent with those previously reported, showing that as the aerobic bacterial genera, they played an important role in the organic nitrogen mineralization during the high temperature period (Zhang et al., 2020; Pan et al., 2023). *Moheibacter*, belonging to the phylum of *Bacteroidetes* and capable of degrading cellulose, was involved in the massive decomposition of organic matters (Wang et al., 2023). Our results showed that *Moheibacter* was revealed with the highest relative abundance at the later stage of composting. *Clostridium_sensu_stricto_1* is ubiquitous in the gastrointestinal tract of animals, generally showing high content in fresh feces. However, previous studies showed that *Clostridium* was not resistant to high temperature, thus the relative content of *Clostridium* was gradually decreased as the composting process was extended (Usui et al., 2017). *Lactobacillus* was widespread in the mammalian gut. Our results showed that as one of the relatively dominant genera during the initial stage of the composting process, the dominant position of *Lactobacillus* was replaced by both genera *Pseudogracilibacillus* (47.07–56.04%) and *Atopostipes* (47.17–88.58%) in the high temperature period of composting.

In summary, the results of relative abundances of bacterial taxa at the phylum and genus levels of each group of composting demonstrated that the addition of illite showed significant effects on the development of bacterial communities during pig manure composting. The presence of illite treated with high temperature increased bacterial diversity and abundance at the end of composting, showing the most significant effects in I-5 group, i.e., the addition of illite could optimize the microbial environment, improve the abundance of microorganisms involved in carbon cycling and nitrogen mineralization, promote the decay process, and accelerate the passivation of heavy metals in the compost.

### 3.5 Effect of different microbial communities and physicochemical properties of the compost heap on Cu and Zn during the composting with the addition of illite

**Figure 6:**
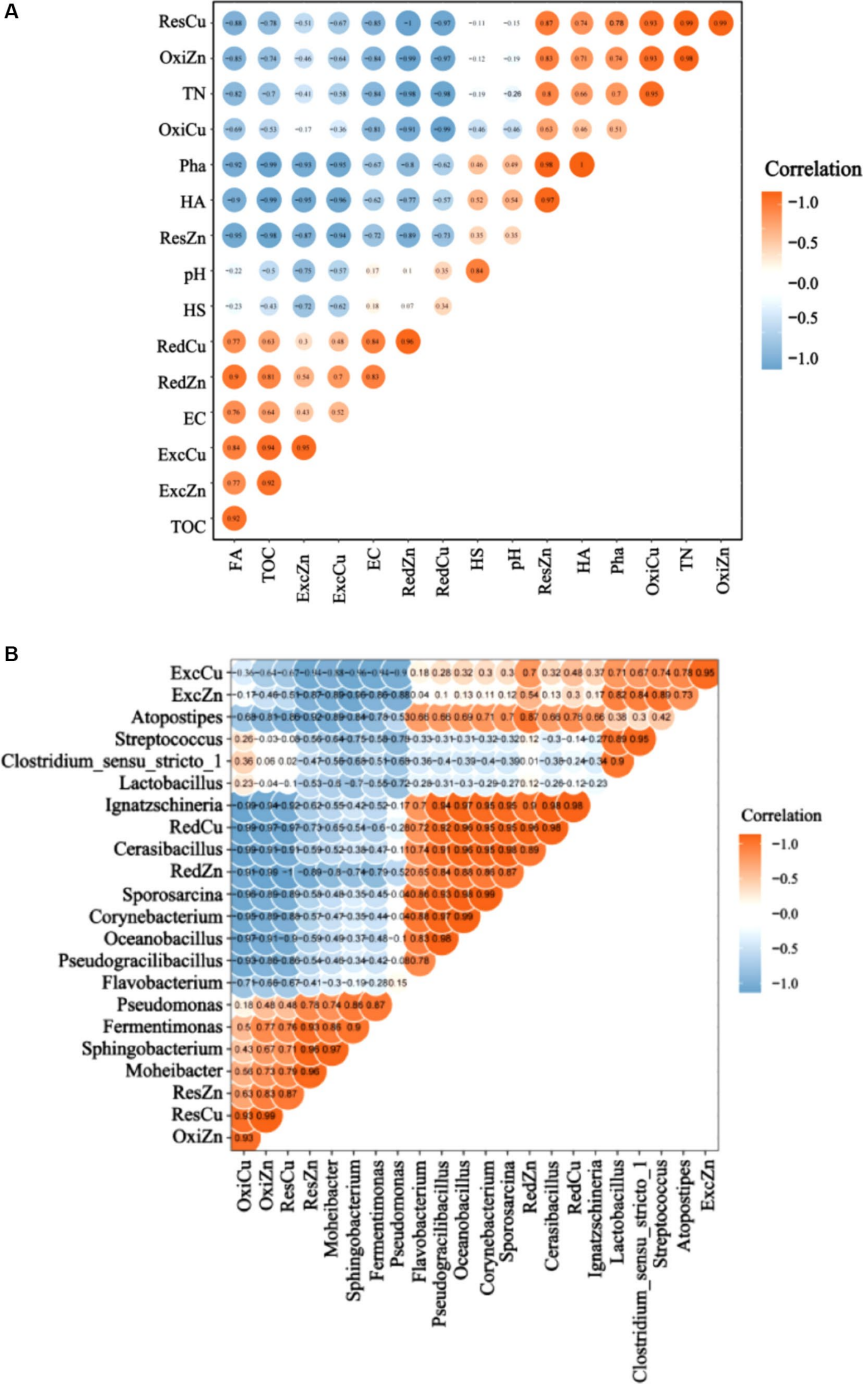
Heatmap analysis of **(A)** physicochemical properties and humification indices and of **(B)** bacterial community succession and heavy metals (Cu and Zn) based on different groups of pig manure composting.

It is well known that composting is an effective way to reduce the bioutilization of heavy metals. Our results showed that the morphological changes of heavy metals during composting were closely related to the physicochemical properties of the compost heap (Figure 6A). These findings were consistent with those previously reported, showing that the physicochemical variations caused by the degradation of organic matters in the composting process could affect the morphological transformation of heavy metals (Amir et al., 2005). The heatmap analysis of various components of heavy metals and physicochemical properties of the compost showed that the change of ExcCu was significantly negatively correlated with both the HA content and Pha (*p* < 0.05), respectively, and significantly positively correlated with the content of FA (p < 0.05). As the process of composting was extended, the binding ability and affinity of HA and ExcCu were gradually increased and the HA promoted the passivation of heavy metals, i.e., ExcCu could easily and tightly bind to humus. Similar to ExcCu, the change of ExcZn was significantly negatively correlated with the content of HA and Pha (p < 0.05), respectively, and significantly positively correlated with the content of FA (p < 0.05), with a large amount of ExcZn chelated with FA in humus. The increased content of RedCu was significantly correlated with HA, FA, TN, Pha, and HA/FA (*p* < 0.05), respectively, whereas RedCu could form complexes with organic acids, thus reducing the bioavailability of Cu. These results were consistent with those previously reported (Wen et al., 2009; Wang et al., 2015). Our results showed that the change of ResCu was significantly negatively correlated with the content of FA and significantly positively correlated with the content of HA and Pha (p < 0.05). These results suggested that the decrease in the bioavailability of Cu and the increase in the contents of OxiCu and ResCu during composting were mainly caused by the conversion of HA to FA. These findings were consistent with those previously reported, showing that the increase of humus content, the change of humus structure (i.e., the increase of aromatic groups), and the stabilization of humus could promote the adsorption complexation between humus and heavy metals (Li et al., 2023). Furthermore, studies have shown that the carboxyl and hydroxyl groups of HA could bind with Zn and Cu ions to form stable complexes, and HA in humus showed a higher molecular weight and a more stable structure compared to FA (Szczerba et al., 2020; Zhao et al., 2022). Therefore, the heavy metals that could generally bind to HA tend to be more stable. In summary, the interconversion between HA and FA promoted the transition of both ExcZn and RedZn to ResZn, ultimately reducing the bioeffectiveness of Zn and Cu in the compost.

In the composting process, the succession of microbial community is closely related to the transformation of heavy metal components. Heatmap correlation analysis was performed based on the microbial communities of compost and the morphological characteristics of heavy metals (Figure 6B). *Moheibacter*, *Sphingobacterium*, *Fermentimonas*, and other bacterial taxa involved in the massive degradation of organic matters were negatively correlated with ExcCu, whereas *Lactobacillus*, *Clostridium_sensu_stricto_1*, and other bacteria with high relative abundances in mammalian fresh feces were positively correlated with ExcZn. RedCu was positively correlated with *Pseudogracilibacillus, Flavobacterium, Pseudomonas, Oceanobacillus, Corynebacterium, Sporosarcina,* and *Cerasibacillus*; these microbes are generally involved in degradation of complex macromolecules and organic nitrogen mineralization, playing an important role in the early stage of composting. RedZn was negatively associated with *Moheibacter, Sphingobacterium,* and *Fermentimonas*, Furthermore, both *Pseudomonas* and *Sphaerobacter* are well known for their high resistance to heavy metals, e.g., *Sphaerobacter* could survive under high Cu environments due to its abundant Cu resistance genes (Qian et al., 2019). These results indicated that the microbes involved in the passivation of heavy metals in compost could affect the passivation of Cu and Zn via promoting the decomposition of organic matters and the formation and transformation of humus. Under the regulation of microorganisms, the humification of organic matters in the process of composting could be promoted by the degradation of carboxyl carbons and the formation of aromatic substances, as previously reported (He et al., 2013). Therefore, it is important to screen the appropriate bacterial strains for the heavy metal passivation in pig manure composting.

**Figure 7:**
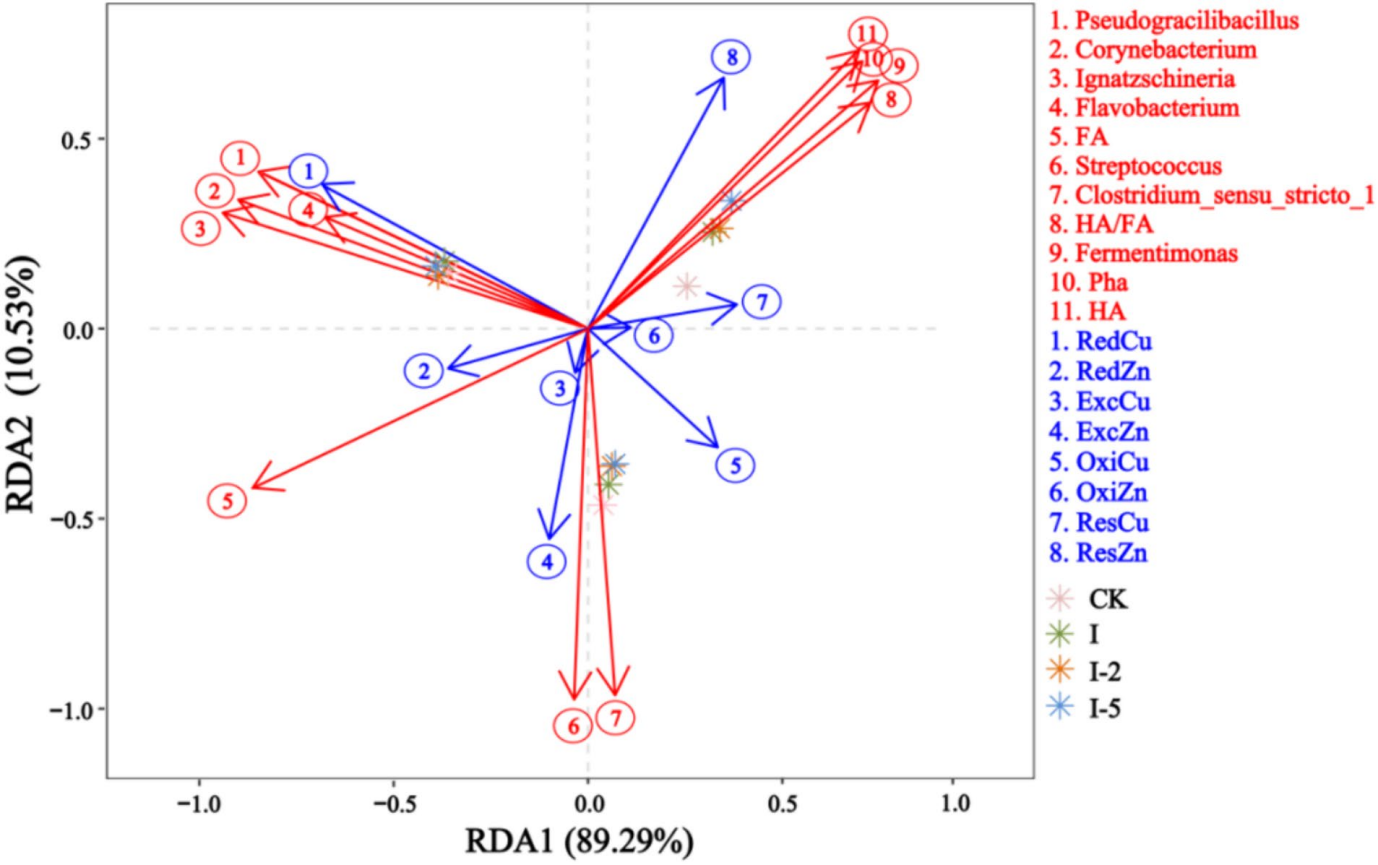
RDA of correlation among core microbial communities, humification, and heavy metals (Cu and Zn) during the pig manure composting process.

In order to clarify the effects of bacterial community and organic component transformation on the variations in the bioavailability of Cu and Zn during the composting with addition of illite, RDA was performed to investigate the correlation among core microorganisms, humification, and bioavailability of heavy metals (Figure 7). The results showed that both ExcCu and ExcZn were positively correlated with the content of FA, *Clostridium*, *Streptococcus*, and *Fermentimonas*, whereas HA and Pha were positively correlated with both ResZn and ResCu, respectively. *Fermentimonas* could degrade the glucose and fructose in the compost and promote the generation of ResCu and ResZn. These results suggested that illite could affect the bioavailability of Zn and Cu via the regulation of the microbial activity and humification of compost.

## 4 Conclusion

Our results showed that the addition of both untreated illite and illite thermally treated under two different temperatures (200 and 500°C) accelerated the degradation of organic matters and increased the temperature and the content of HA in the compost. Compared with the control group, humification was promoted in the illite treatment groups. The passivation of Zn and Cu was positively correlated with humification, the content of HA, and Pha, and negatively correlated with the content of FA, while the addition of illite increased the passivation of Zn and Cu by promoting the humification process. The highest passivation levels of Cu and Zn and the highest levels of humification and maturity of the composting were obtained in the I-5 treatment group. In summary, use of appropriate passivation agent in composting could promote the microbial metabolism, accelerate the composting, and promote the chelation between heavy metal ions and organic matters, ultimately improving the passivation and reducing the bioavailability of heavy metals in compost.

## Data availability statement

The datasets presented in this study can be found in online repositories. The names of the repository/repositories and accession number(s) can be found in the article/supplementary material.

## Author contributions

ME: Writing – review & editing, Formal analysis, Investigation, Visualization, Writing – original draft. JJ: Visualization, Writing – review & editing, Conceptualization, Formal analysis, Investigation, Writing – original draft. YW: Data curation, Writing – review & editing, Conceptualization, Methodology. MF: Writing – review & editing, Data curation, Visualization. CB: Writing – review & editing, Data curation, Software, Visualization. JM: Writing – review & editing, Validation. SC: Writing – review & editing, Methodology. SZ: Writing – review & editing, Visualization. JZ: Funding acquisition, Writing – review & editing. WZ: Data curation, Writing – review & editing. GC: Funding acquisition, Project administration, Writing – review & editing. HC: Conceptualization, Writing – review & editing, Resources.
